# Wearable Reduced-Channel EEG System for Remote Seizure Monitoring

**DOI:** 10.3389/fneur.2021.728484

**Published:** 2021-10-18

**Authors:** Mitchell A. Frankel, Mark J. Lehmkuhle, Mark C. Spitz, Blake J. Newman, Sindhu V. Richards, Amir M. Arain

**Affiliations:** ^1^Epitel, Inc., Salt Lake City, UT, United States; ^2^Neurology, University of Colorado Anschutz Medical Center, Aurora, CO, United States; ^3^Department of Neurology, University of Utah School of Medicine, Salt Lake City, UT, United States

**Keywords:** seizure detection, wearables, remote monitoring, machine learning, EEG

## Abstract

Epitel has developed Epilog, a miniature, wireless, wearable electroencephalography (EEG) sensor. Four Epilog sensors are combined as part of Epitel's Remote EEG Monitoring platform (REMI) to create 10 channels of EEG for remote patient monitoring. REMI is designed to provide comprehensive spatial EEG recordings that can be administered by non-specialized medical personnel in any medical center. The purpose of this study was to determine how accurate epileptologists are at remotely reviewing Epilog sensor EEG in the 10-channel “REMI montage,” with and without seizure detection support software. Three board certified epileptologists reviewed the REMI montage from 20 subjects who wore four Epilog sensors for up to 5 days alongside traditional video-EEG in the EMU, 10 of whom experienced a total of 24 focal-onset electrographic seizures and 10 of whom experienced no seizures or epileptiform activity. Epileptologists randomly reviewed the same datasets with and without clinical decision support annotations from an automated seizure detection algorithm tuned to be highly sensitive. Blinded consensus review of unannotated Epilog EEG in the REMI montage detected people who were experiencing electrographic seizure activity with 90% sensitivity and 90% specificity. Consensus detection of individual focal onset seizures resulted in a mean sensitivity of 61%, precision of 80%, and false detection rate (FDR) of 0.002 false positives per hour (FP/h) of data. With algorithm seizure detection annotations, the consensus review mean sensitivity improved to 68% with a slight increase in FDR (0.005 FP/h). As seizure detection software, the automated algorithm detected people who were experiencing electrographic seizure activity with 100% sensitivity and 70% specificity, and detected individual focal onset seizures with a mean sensitivity of 90% and mean false alarm rate of 0.087 FP/h. This is the first study showing epileptologists' ability to blindly review EEG from four Epilog sensors in the REMI montage, and the results demonstrate the clinical potential to accurately identify patients experiencing electrographic seizures. Additionally, the automated algorithm shows promise as clinical decision support software to detect discrete electrographic seizures in individual records as accurately as FDA-cleared predicates.

## Introduction

Epilepsy affects 1% of the population or ~70 million people worldwide (1). For people who are experiencing seizure-like activity in their daily lives, the current acceptable method for differential diagnosis requires a visit to an epilepsy monitoring unit (EMU), for which there only exists ~245 Level III/IV centers out of ~6,200 hospitals across the U.S (2). Most commonly, this requires a limited multi-day stay in the EMU where video and high-channel-count wired electroencephalography (EEG) are recorded (19+ EEG channels). A diagnosis of epilepsy is determined only after epileptologist review of the video-EEG record for electrographic epileptiform events and clinical seizure activity. EMU visits require time away from home, potentially large travel distances, can be very costly even with insurance, require restricted movements due to the wired and tethered EEG systems, and can be traumatizing (3, 4). During these limited EMU stays, adults are commonly taken off their medications with the intent to record seizures. It is common for some people to feel overwhelmed by the entire process and leave early without any recording of seizures or concrete diagnosis of a seizure disorder (4). Additionally, many people do not have any electrographic seizure activity during their EMU stay for various reasons including the rarity of events that do not occur during a limited EMU stay (3, 5).

**Figure 1:**
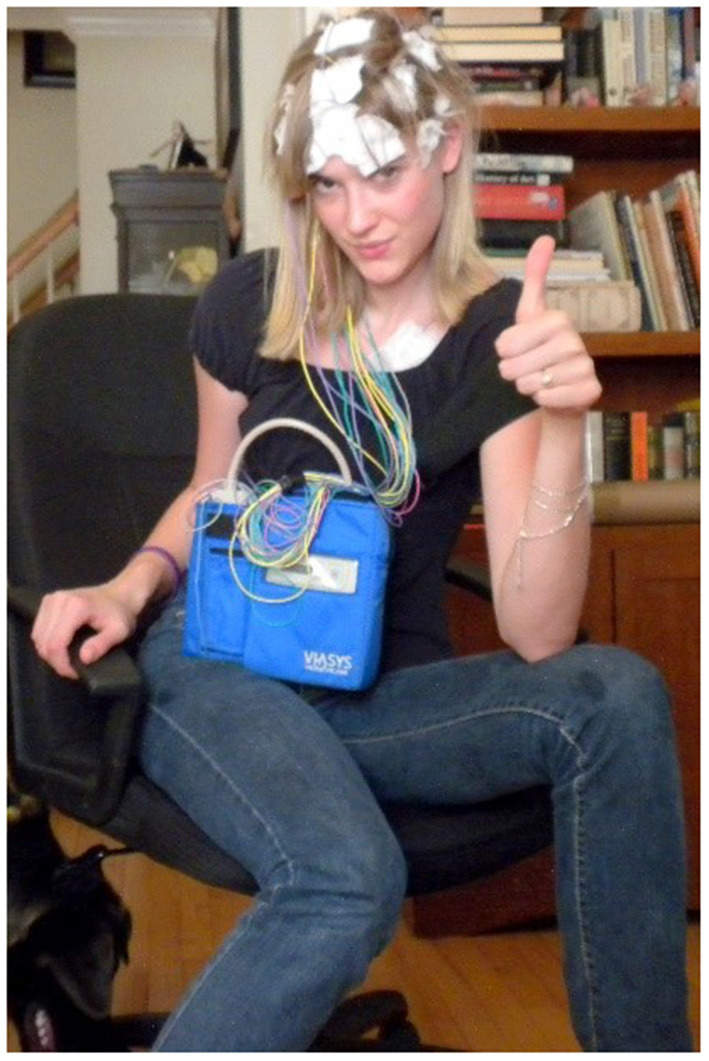
Typical wired ambulatory EEG (with permission from Kelly Falk).

Better electrographic seizure recordings and clinical decisions could be made if the EEG was recorded at home in a person's normal daily environment (6). Ambulatory EEG systems (AEEG) have been used by epileptologists for diagnostic purposes when an in-EMU visit is not possible. The AEEG systems allow people to wear the wired AEEG in their home environment, but have substantial limitations including: the EEG electrodes must be positioned and glued to the person's scalp by a trained EEG technician, the long wired tethers create obfuscating motion artifacts in the EEG record especially during convulsive seizures and other movements, the bulky system is restrictive which prevents the person from many normal daily activities like exercise or bathing, and the cumbersome system can be socially stigmatizing if worn out in public (Figure 1) (7, 8). Sub-scalp EEG systems, such as UNEEG's SubQ (9) and EpiMinder's Minder™ (10) offer potential solutions for long-term, at-home EEG recording, though they are invasive. A discreet, wireless, easily applied, wearable EEG system would have the potential to make home EEG recording more widely available, less restrictive, and provide EEG with no wired tether artifacts that can obscure the electrographic seizure activity.

**Figure 2:**
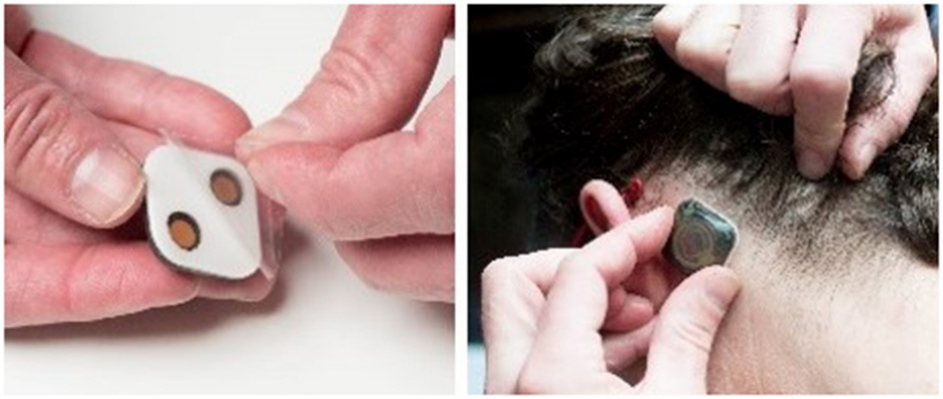
Epilog uses one-piece disposable “stickers,” that are both the adhesive and conductive hydrogel that serve as the interface between Epilog and the scalp when used below hairline.

To fill this need, Epitel has developed Epilog (Figure 2), a miniature, wireless, wearable EEG sensor capable of recording high-fidelity EEG throughout a person's daily life. Epilog is smaller than a cochlear implant, is designed to be aesthetically-pleasing while worn on the scalp below the hairline, and allows unrestricted mobility. The Epilog sensor records a single channel of EEG through a differential electrode pair spaced 18 mm center-to-center, similar to high-density EEG (11). Because all components are self-contained, Epilog data is less susceptible to wired movement artifacts or antenna noise that plague wired EEG systems. Epilog sensors are robust, water-resistant, and designed to meet the rigorous needs of everyday use while providing consistent recording performance.

**Figure 3:**
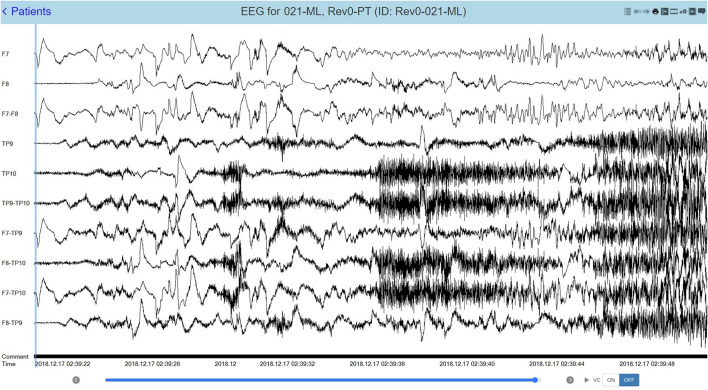
A 30-s recording from four Epilog sensors during a focal-onset seizure evolving to bilateral tonic-clonic (TP10 focal onset), displayed in the 10-channel REMI montage.

Epitel has developed REMI®, a Remote EEG Monitoring platform. REMI is intended to be used in any clinical situation where near real-time and/or remote EEG is warranted, using four Epilog sensors placed bilaterally below the hairline on the forehead and behind the ear, at the approximate F7/F8 and T5/T6 locations based on the standard International 10–20 system (12). Epitel intends to extend the use of REMI for ambulatory, outpatient EEG recordings, where any physician can prescribe the system for a person suspected of seizures. Their patient would wear the sensors for a specific prescribed duration with no mobility restrictions in their normal daily lives. The EEG data from the four Epilog sensors are directly uploaded to an HIPAA-compliant database, converted into a 10-channel REMI montage that includes the four individual sensor recordings and six sensor-to-sensor differential channels (herein referred to as “REMI montage”). The REMI montage is accessible for review by a remote epileptologist through REMI's Persyst® Mobile interface. An example of a 30-s focal onset seizure evolving to bilateral tonic-clonic recorded and presented in the REMI montage can be seen in Figure 3.

Reviewing long duration EEG recordings can be a very time-consuming processes for epileptologists and having more at-home EEG recordings via wearable systems, such as REMI, would likely become overly burdensome. Automated seizure detection algorithms may be used as Clinical Decision Support Software (CDSS), where the algorithm highlights specific time periods in an EEG record when seizure activity is likely. Epileptologists could use the markers to guide and speed up their EEG review. A wide variety of signal processing and machine learning algorithms have been studied for seizure detection purposes (13–16). There are clinically cleared CDSS that reduce the time required to review EEG in the hospital with high sensitivity and low false detection rates (FDR) (17–23). The most commonly used clinically-cleared software for all types of seizure detection is Persyst®. The Persyst 12 software is the most commonly cited predicate for scalp EEG seizure detection software with a mean sensitivity of 81% and FDR of 0.21 false detections per hour. However effective, automated scalp EEG seizure detection software is currently only clinically available for use on in-hospital, high-channel-count, wired-EEG recordings.

To demonstrate feasibility for long-term ambulatory use of REMI, it is necessary to first determine how accurate remote epileptologist reviewers are at identifying spontaneous, recurrent, electrographic seizures in the REMI montage, and if automated algorithms can be used as CDSS to support expert review. With this study, we hypothesize that (a) Epileptologists can accurately detect focal-onset electrographic seizures in REMI montage data, (b) Automated seizure detection algorithms can be used to detect focal-onset electrographic seizures in REMI montage data with sensitivity and false detection rate similar to FDA-cleared predicates, and (c) Automated seizure detection algorithms can be used as CDSS to guide epileptologist review without a loss in performance.

## Methods

Electroencephalography was recorded by Epilog sensors alongside standard-of-care 19-channel, full-montage, video-EEG (herein referred to as “wired-EEG”) in adults during EMU stays at the University of Colorado Anschutz Medical Center. The subjects' wired-EEG included a full array of 19 wired electrodes in the standard International 10–20 system, including T1, T2, and eye leads.

### General Methods

**Figure 4:**
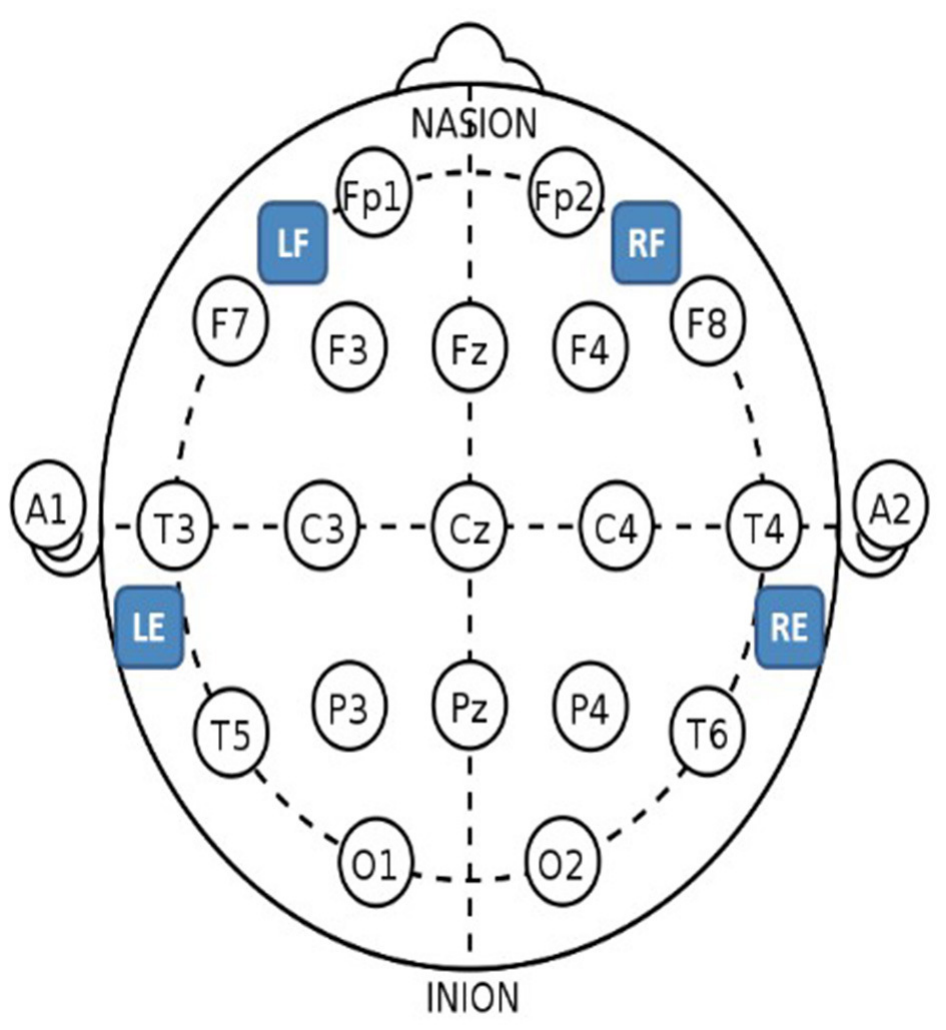
Epilog sensor placement locations with the standard International 10–20 system as reference. For the forehead locations, LF is left-forehead closest to F7, RF is right-forehead closest to F8, and the Epilog sensors were placed as far from forehead/eye muscles as possible. For the behind-the-ear locations, LE is behind left ear closest to T5, and RE is behind right ear closest to T6, and the Epilog sensors were placed as high up as possible over the mastoid while still being below the hairline, making sure placement was not directly over the neck muscles.

All protocols were approved by the Institutional Review Board of the University of Colorado. Adults entering the EMU for long-term EEG evaluation were called prior to their appointment to discuss the study objectives. Each subject was consented in the EMU. Epilog sensors were placed by the trained study coordinator after the full-montage wired-EEG electrodes were affixed by an EEG technician. Each subject wore four Epilog sensors, placed at scalp locations below the hairline on the forehead and behind each ear, using an adhesive sticker with embedded conductive hydrogel (see Figure 2 for hydrogel sticker and see Figure 4 for sensor proximity to 10–20 locations). The IRB approval allowed for up to 7 days of continuous EEG recording. The Epilog sensor can be worn continuously for up to 7 days in a normal EMU environment and required no daily maintenance from the subject or medical staff. Routine video-EEG review and associated seizure identification was part of the standard patient care.

### EEG Recordings

Epilog records a single channel of EEG through two gold electrodes Ø6 mm, spaced 18 mm center-to-center, and data is extracted from the sensor's onboard memory into the European Data Format Plus file type (EDF+). Epilog data were recorded at 10-bit, 512 Hz with an amplifier passband of 0.8–92 Hz. The full-scale signal amplitude was ± 175 μV. The Epilog sensor uses a primary battery that supports continuous EEG recording for 7 days without replacement or recharging. Video-EEG in the EMU was recorded with standard clinical equipment and settings (Nihon Khoden Neurofax EEG-1200, 200 Hz sampling).

### Workflow

The study coordinator working with the on-service epileptologist pre-contacted all subjects, consented all subjects upon arrival, placed each Epilog sensor after the EEG Technician had placed wired-EEG electrodes (using training material provided by Epitel), and managed the reporting and data retrieval. The Epilog EEG and wired-EEG were time-synced using a sequence of “taps” on both the Epilog sensor and the Fp1 wired-EEG electrode. During standard-of-care video-EEG review, the epileptologist determined the electrographic start and stop time for each seizure event. The clinical data management specialist then uploaded de-identified subject data and Epilog EEG records to a HIPAA-compliant database. The EEG data from the four Epilog sensors was converted into the 10-channel REMI montage (Figure 3) and uploaded into a Persyst 14b server with Persyst Mobile access.

### Automated Seizure Detection Algorithm

Prior to designing the algorithm for this study, Epitel began developing a seizure detection algorithm for single-channel EEG recorded with Epilog sensors. Epilog EEG data from patients at clinical partner epilepsy centers were used to determine which specific features, machine learning model types, and machine learning model hyperparameters are most likely to yield the best results for focal seizure detection in Epilog sensor EEG. This was done using common grid search, feature importance, and stratified k-folds cross-validation methods. This early analysis can be considered the train-test data for feature and model determination. The single-channel Epilog EEG data used in the train-test design of the algorithm was from patients who did not wear Epilog sensors at all four scalp locations as in this present study. While the design of the single-channel algorithm is ongoing, the preliminary knowledge supported the use of the specific features and machine learning model and parameters used in the present study.

EEG data is commonly separated into short, seconds-long segments for seizure detection algorithms that use machine learning (15, 16). The 10-channel REMI-montage data was segmented into 2-s windows for scoring and feature extraction. Data segments that occurred during known electrographic seizure times were scored as “ictal” segments; segments that occurred within 15 min before and after known seizure times were scored “near-ictal”; and all other segments were scored “non-ictal.” The reasoning behind the “near-ictal” scoring is that there was some pre-ictal evolution in the EEG prior to the noted electrographic onset as well as some post-ictal EEG activity that might confound a machine learning classifier. The 15-min “near-ictal” timing was chosen based on internal review of the Epilog EEG data and knowledge that no known seizure event timings were within <15 min of each other. For each 2-s segment, features were extracted in the time domain (e.g., variance), frequency domain (e.g., power in delta band), time-frequency domain (e.g., wavelet convolutions), and complexity domain (e.g., entropy). Because seizures are known to evolve over time, historical information about each feature was determined as a weighted average of prior segments' feature values and added to the overall feature set. Cross-channel correlations for all features were determined and added to the overall feature set for each 2-s segment.

For each of the 20 subjects, a training feature set was created by combining the segmented and scored features from all other 19 subjects, essentially a leave-one-out method that occurs 20 independent times. The ratio of non-ictal to ictal data was quite high, even for those who experienced seizures, which would bias any machine learning classifier. Thus, the training set was reduced to keep all of the ictal segments and randomly chosen non-ictal segments in a 3:1 non-ictal:ictal ratio. No “near-ictal” segments were included in the training set. A random forest machine learning classifier was trained using an ensemble of 500 trees, where bootstrapping (sample with replacement) was allowed, and trees were extended to full splits. The trained classifier was then applied to the held-out subject's complete feature set including “near-ictal” segments, and an ictal class likelihood was determined for each 2-s segment.

In most literature, this is when a threshold is applied to classify each segment as ictal or not, and metrics such as receiver operating characteristic curve area-under-the-curve or F1-score can be determined to see how well the classifier performed in out-of-sample segmented data. For seizure detection, this is not enough, as what is necessary is whole seizure start and stop times, where a complete seizure can be between 10 s and 15 min in duration. This has been demonstrated in some recent literature using the classifier likelihood output and an integrate-and-fire neuron (24). For our algorithm, a leaky, weighted integrator was applied to the classifier likelihood output. Using a fixed threshold for all subjects, when the integrated ictal likelihood went above the threshold for five continuous segments (10 s), a seizure start marker was set. The exact time point of the start marker was set based on the start time of the first segment above threshold and accounted for the duration of the integrator. Similarly, when the integrated ictal likelihood went below the threshold for five continuous segments, a seizure stop marker was set. This threshold was set low so that the sensitivity of the machine learning algorithm would be high (≥90% on the training data) at the expense of a possibly high FDR. Some additional processing was done on these whole-seizure detection events: (1) Any events that occurred within 2 min of each other were concatenated into a single event, and (2) After the concatenation, any event that lasted longer than 15 min was discarded. The sensitivity, precision, and FDR of the automated algorithm were determined for each of the 20 subjects, where a true positive (TP) event occurs if there is any overlap between the known the seizure electrographic onset/offset time and the algorithm determined one (25). For TP events, the percent of overlap was determined as the amount of time of the known seizure event that the detection event encompassed.

### Blinded Expert Review With Persyst Mobile

Three independent, board certified epileptologists not affiliated with University of Colorado Anschutz Medical Center, and who have never reviewed Epilog EEG in the REMI montage before, were recruited for this study. Each epileptologist was provided the REMI montage from 40 subjects who wore four Epilog sensors, to remotely review through Persyst Mobile. The 40 records consisted of randomized data from: (a) unannotated EEG from 10 subjects who had focal-onset electrographic seizures during their EMU stay, (b) unannotated EEG from 10 subjects who had no electrographic seizures or epileptiform activity during their EMU stay, (c) algorithm-determined seizure-detection start/stop annotated EEG from the same 10 subjects who had focal-onset electrographic seizures during their EMU stay, and (d) algorithm-determined seizure-detection start/stop annotated EEG from the same 10 subjects who had no electrographic epileptiform activity during their EMU stay. The epileptologists were blinded to (a) how many of the 40 subjects were known to have seizures, (b) that the same 20 subjects' EEG was the data that was processed to create the algorithm-determined annotated EEG, and (c) the randomized order that the data was presented in. There were no algorithm-determined seizure events for some of the 10 subjects who did not have electrographic activity, and thus no annotations in the EEG record. Because of this, the 20 EEG records where automated seizure detection was applied were noted in their subject ID with “ML,” visible in Persyst Mobile. As far as the epileptologists knew, there were 40 independent data sets, some of which contained focal-onset seizures, and 20 of which had CDSS algorithm-determined events annotated.

The epileptologists were asked to review the non-ML records in their entirety and annotate any sections of the EEG data that they believed to be indicative of electrographic seizure activity. The epileptologists were also asked to review the algorithm-annotated records and annotate electrographic seizure activity, using the algorithm-determined events as CDSS. The reviewers were told that these records were processed with an automated seizure-detection algorithm that was tuned so that the sensitivity would be high across all subjects with possibly high FDR. The reviewers were told to use their judgement as to how much they relied on the algorithm annotations or lack thereof. A majority consensus (best 2 out of 3) was used due to the well-known inter-rater variability in blinded EEG review, even among expert neurologists (26). Sensitivity, precision, and FDR [false positives per hour (FP/h)] were determined for each subject for both algorithm-annotated and unannotated records, using the “any-overlap” method discussed earlier. For TP events, the percent of overlap was determined using the method previously discussed. For all known seizure times, the inter-rater reliability was measured with Cohen's Kappa for pair-wise reviewers and Fleiss' Kappa for group reliability.

## Results

**Figure 5:**
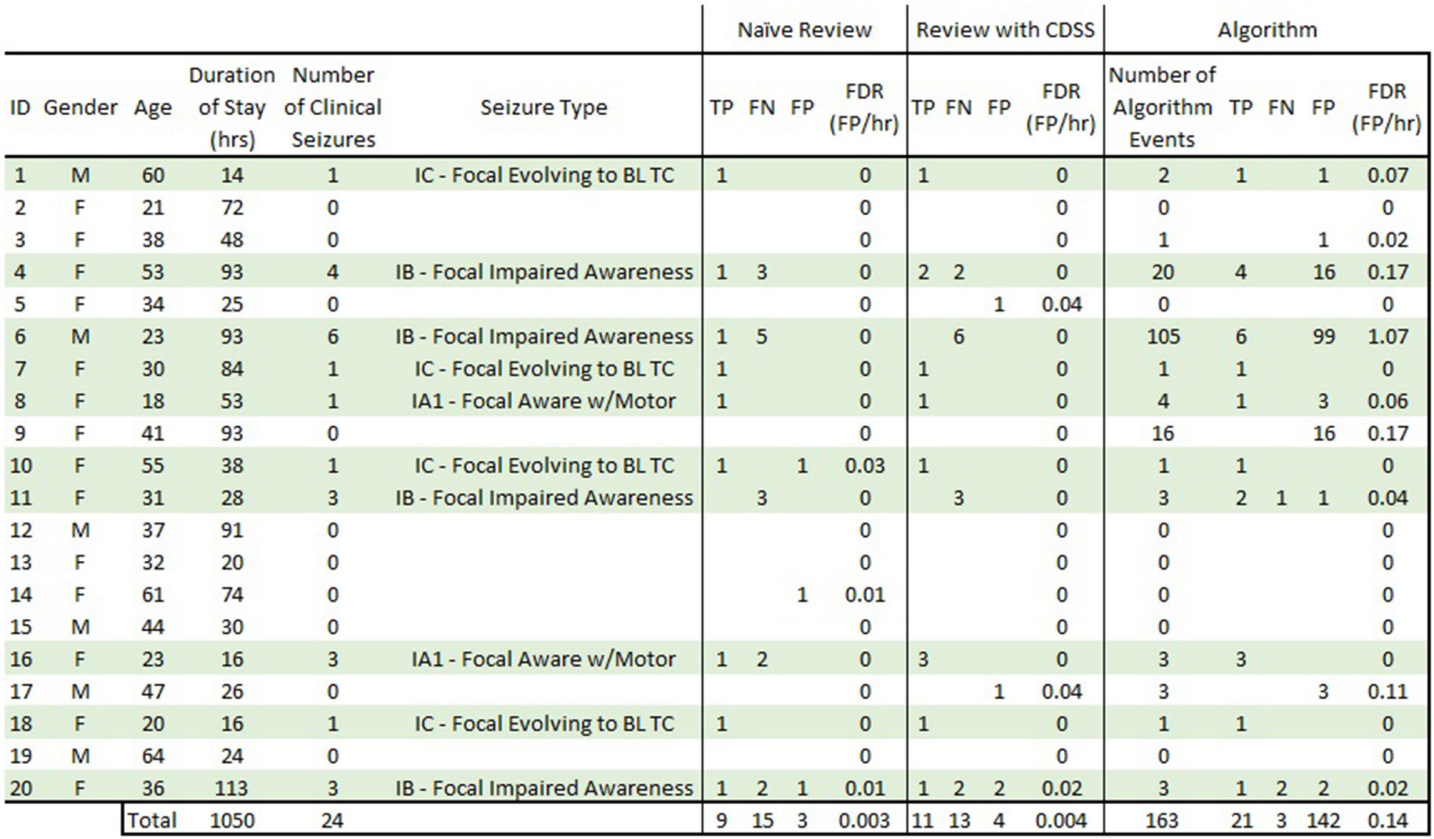
Complete subject demographics and results. Subjects in light green are those that had focal-onset seizures during their EMU stay. “Naive Review” results are consensus epileptologist review of the REMI montage without algorithm-detection annotations. “Review with CDSS” results are consensus epileptologist review with algorithm-detection annotations. “Algorithm” results are for the REMI automated seizure detection algorithm. TP, true positive; FN, false negative; FP, false positive; FDR, false detection rate; BL TC, bilateral tonic-clonic.

As part of a larger study, a total of 40 adult subjects (ages 18–64) were enrolled, and 22 (55%) had at least one seizure in the EMU. For this specific piece of the study, data from 20 subjects (ages 18–64) were used: 10 subjects who had focal-onset seizures as classified according to ILAE (27) and 10 subjects who were determined to have no seizure events or epileptiform activity in their wired-EEG (Figure 5). A total of 24 focal onset seizures were recorded in the 10 subjects (min 1, mean 2.4, median 2, max 6). The 20 subjects had EMU stays between 0.5 and 5 days (mean of 2.2 days). Figure 5 details the demographics and results for all subjects. Table 1 is a summary of sensitivity, precision, and FDR. Table 2 is a summary of the inter-rater reliability. Table 3 is a summary of the results based on seizure type. Table 4 is a summary of the overlap between known seizures and reviewer- and algorithm-detected TP seizures.

**Table 1:** Summary of sensitivity, precision, and false detection rate results.

**Reader**	**Sensitivity**	**Precision**	**False detection rate**
	**% ± SD [range]**	**% ± SD [range]**	**FP/h ± SD [range]**
R1—Naive	58 ± 46 [0–100]	75 ± 42 [0–100]	0.006 ± 0.016 [0.0–0.062]
R2—Naive	57 ± 47 [0–100]	58 ± 47 [0–100]	0.031 ± 0.044 [0.0–0.109]
R3—Naive	73 ± 40 [0–100]	78 ± 37 [0–100]	0.006 ± 0.013 [0.0–0.038]
**Consensus—Naive**	**61 ± 42 [0–100]**	**80 ± 35 [0–100]**	**0.002 ± 0.007 [0.0–0.026]**
R1—CDSS	58 ± 47 [0–100]	70 ± 48 [0–100]	0.007 ± 0.019 [0.0–0.076]
R2—CDSS	53 ± 50 [0–100]	42 ± 44 [0–100]	0.020 ± 0.054 [0.0–0.244]
R3—CDSS	75 ± 41 [0–100]	73 ± 37 [0–100]	0.010 ± 0.020 [0.0–0.072]
**Consensus—CDSS**	**68 ± 43 [0–100]**	**73 ± 44 [0–100]**	**0.005 ± 0.012 [0.0–0.040]**
**Algorithm**	**90 ± 22 [33–100]**	**60 ± 38 [6–100]**	**0.087 ± 0.238 [0.0–1.069]**

*Naïve results are for epileptologist review without algorithm detection support. CDSS results are for epileptologist review with algorithm detection support. Algorithm results are for the REMI automated seizure detection algorithm (SD, standard deviation; FP, false positive)*.

**Table 2:** Inter-rater reliability for known seizure events.

	**Naive**	**CDSS**
R1 vs. R2	0.71	0.44
R1 vs. R3	0.59	0.53
R2 vs. R3	0.52	0.53
Group	0.59	0.49

*The values shown are Cohen's Kappa statistic for pair-wise (e.g., Reviewer R1 vs. Reviewer R2) and Fleiss' Kappa statistic for the group*.

**Table 3:** Electrographic seizures ending in a convulsion (convulsive) vs. seizures that did not end in a clinical convulsion (non-convulsive).

	**Naive review**				**Review with CDSS**				**Algorithm**	
	**Consensus**		**All 3**		**Consensus**		**All 3**			
	**TP**	**FN**	**TP**	**FN**	**TP**	**FN**	**TP**	**FN**	**TP**	**FN**
Conv.	8		5		6	2	6		8	
Non-conv.	3	13		10	3	13		11	13	3

*The Consensus review is best 2 out of 3 and the “All 3” is when all three reviewers agree. The consensus, all three reviewers, and the algorithm all performed better for detecting seizures that ended in a convulsion. TP, true positive; FN, false negative*.

**Table 4:** Percent of overlap between known seizures and true positive events marked by reviewers and the automated algorithm.

	**Min**	**Max**	**Mean**	**SD**
R1—Naive	43	96	80.8	17.3
R2—Naive	54	100	83.5	18.2
R3—Naive	48	100	86.1	15.6
R1—CDSS	64	100	87.2	11.9
R2—CDSS	63	100	90.6	12.5
R3—CDSS	49	100	85.1	15.4
Algorithm	44	100	92.1	13.8

*SD, standard deviation*.

### Blinded Consensus Epileptologist Review of Unannotated Data

**Figure 6:**
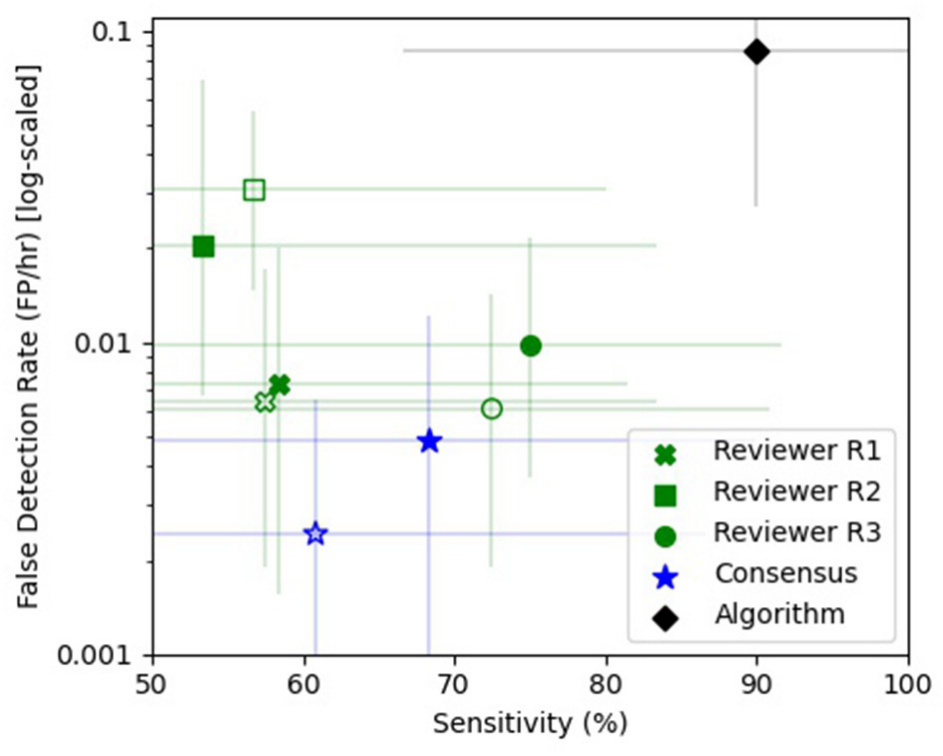
Sensitivity vs. false detection rate. The sensitivity (%) and false detection rate (FDR—FP/h) are shown for the individual reviewers [green x (R1), square (R2), and circle (R3)], the consensus review (blue star), and the automated algorithm (black diamond). The unfilled markers denote the naive epileptologist review and the filled markers denote review with automated algorithm annotations as clinical decision support software (CDSS). Reviewers R1 (green x), R3 (green circle), and the consensus (blue star) showed improved sensitivity with CDSS at the expense of a higher FDR, while reviewer R2 (green square) had a reduced sensitivity and FDR with CDSS. The REMI automated seizure detection algorithm (black diamond) had a higher sensitivity than any one reviewer or consensus, with a higher false detection rate. The error bars are 95% confidence intervals using a bias-corrected and accelerated method (BCa, *N* = 1,000).

The consensus review identified at least one electrographic seizure for 9 out of the 10 subjects who had known electrographic seizures during their EMU stay (90% Sensitivity) and identified no false events for 9 out of the 10 subjects who had no electrographic seizures during their EMU stay (90% Specificity). Consensus detection of individual electrographic seizures resulted in a mean sensitivity of 61% across the 10 subjects (100% for 5 out of 10 subjects, Figure 5—Naive Review, Figure 6—unfilled blue star, Table 1). The range of the per-reviewer mean sensitivity was 57–73% (Table 1; Figure 6—unfilled green markers). The consensus mean precision, or positive predictive value (PPV), was 80% across all subjects, with a per-reviewer mean PPV range of 58–78%. The consensus mean FDR was 0.002 FP/h of data, with a per-reviewer mean FDR range of 0.006–0.031 FP/h. The inter-rater reliability (Table 2) ranged from 0.52 (moderate) to 0.71 (good) for the pair-wise comparison as measured with Cohen's Kappa statistic and 0.59 (moderate) for the group as measured with Fleiss' Kappa statistic. The consensus review was able to accurately mark 8 out of 8 focal onset seizures that ended in a clinical convulsion (Type IA1—Focal Aware w/Motor and Type IC—Focal Evolving to Bilateral Tonic Clonic) with all three reviewers marking 5 of 8 (Table 3). The minimum overlap between known seizures and TP reviewer determined events ranged from 43 to 54%, with means ranging 80.8–86.1% (Table 4).

### Blinded Consensus Epileptologist Review With Algorithm-Detection as CDSS

When the reviewers were provided algorithm seizure-detection annotations in the EEG record as CDSS, the consensus review identified at least one electrographic seizure for 8 out of the 10 subjects who had known electrographic seizures during their EMU stay (80% Sensitivity) and identified no false events for 8 out of the 10 subjects who had no electrographic seizures during their EMU stay (80% Specificity). The mean sensitivity for consensus detection of individual electrographic seizures improved to 68% across the 10 subjects (100% for 6 out of 10 subjects, Figure 5—Review with CDSS, Figure 6—filled blue star; Table 1). The range of the per-reviewer mean sensitivity was 53–75% (Table 1; Figure 6—filled green markers). The consensus mean precision (PPV) was reduced to 73% across all 20 subjects, with a per-reviewer mean PPV range of 42–73%. The consensus mean FDR increased to 0.005 FP/h, with a per-reviewer mean FDR range of 0.007–0.020 FP/h. The inter-rater reliability was reduced (Table 2) and ranged from 0.44 (moderate) to 0.53 (moderate) for the pair-wise comparison as measured with Cohen's Kappa statistic and 0.49 (moderate) for the group as measured with Fleiss' Kappa statistic. The consensus review accurately marked 6 out of 8 focal onset seizures that ended in a clinical convulsion (Type IA1—Focal Aware with Motor and Type IC—Focal Evolving to Bilateral Tonic Clonic) with all three reviewers marking 6 of 8 (Table 3). The minimum overlap between known seizures and TP reviewer determined events ranged from 49 to 64%, with means ranging 85.1–90.6% (Table 4).

### Automated Seizure Detection

Epitel's automated seizure detection algorithm was able to detect 21 of 24 known focal-onset seizures (out-of-sample), providing a mean sensitivity of 90% across all subjects (100% for 8 out of 10 subjects, Figure 5—Algorithm, Figure 6—filled black diamond; Table 1). The three missed events were all Type IB—Focal Onset with Impaired Awareness (Table 3). The mean precision (PPV) of the algorithm was 60% and the mean FDR was 0.087 FP/h across all subjects. The FDR was 0.22 FP/h for subjects who had electrographic seizures during their EMU stay and 0.04 FP/h for subjects who did not have seizures. Subject #6 had an outlier FDR of 1.07 FP/h (99 false detections during the 93-h EMU stay), while the maximum FDR for all others was 0.17 FP/h. The algorithm mean sensitivity was higher than any individual reviewer or consensus review, with or without CDSS, but with a higher FDR (Figure 6). The algorithm detected at least one TP seizure event for all 10 subjects who had electrographic seizures (100% sensitivity) and detected no false positives (FP) for 7 of the 10 subjects who had no seizures (70% specificity). The number of FP and FDR for the other three non-seizure subjects was one event, 0.02 FP/h (ID #3), 16 events, 0.17 FP/h (ID #9), and three events, 0.11 FP/h (ID #17). The minimum overlap between known seizures and TP algorithm-determined events was 44% with a mean of 92.1% (Table 4).

## Discussion

This study sought to answer if epileptologists could accurately detect focal-onset electrographic seizures with a remote, wireless, reduced-channel EEG system, and whether automated algorithms could assist with that task.

### Blinded Epileptologist Review of Unannotated Data

This is the first study to analyze epileptologists ability to blindly review remote EEG data from four Epilog sensors in the 10-channel REMI montage. There are only a few studies assessing the performance of individual reviewers and consensus review for blind seizure identification in EEG. In a large ICU study using standard clinical wired-EEG, Tu et al. reported a consensus sensitivity of 75% and individual reviewer FDR of 0.085 FP/h (26). Other studies have shown individual reviewer sensitivities between 70 and 85% with FDR between 0.016 and 0.043 FP/h (20). Epileptologists are trained on, and are most commonly experienced with, reviewing 19+ channels of video-EEG in multi-channel montages. Therefore, it is not surprising that they might have some difficulty with the 10-channel REMI montage, especially on their first experience. With no prior training or experience with Epilog EEG in the REMI montage, the reviewer consensus successfully achieved a sensitivity of 61%, precision (PPV) of 80%, and FDR of 0.002 FP/h for identifying spontaneous recurrent electrographic focal-onset seizures (Figure 6; Table 1). As might be expected, the reviewers were very conservative in marking seizures, including a very low FDR when compared to prior studies. While the reviewers had difficulty determining focal-onset seizures that did not result in a clinical convulsion, they were very good at detecting events that did end in a clinical convulsion where the consensus review found all eight events with all three reviewers finding 5 of the 8 (Table 3). The overlap between known seizure timings and reviewer-determined events was generally high, with means above 80% (Table 4).

### Blinded Epileptologist Review With CDSS Annotations

The reviewers were also provided with randomized copies of the REMI montage records with annotations of seizure event markers determined by an automated algorithm with high sensitivity. The consensus review improved from 61 to 68% on these records, with a slight increase in FDR (0.005 FP/h up from 0.002 FP/h) (Table 1). While the results are an improvement, they are not statistically different (Figure 6). The reviewers were only told that the algorithm had a high sensitivity with a possible high FDR, but not what the exact values would be, and no training was provided on how to use the algorithm event annotations as CDSS. During a post-analysis debrief, the reviewers mentioned they evaluated the EEG in its entirety and then checked their review against the algorithm-detected events for overlap. The reviewers felt the algorithm “overcalled” events. There were a high number of algorithm-detected FPs for three subjects (4, 6, and 9). Two of the four TP markers and none of the 16 FP markers for Subject 4 were noted by the consensus review, an improvement of 1 TP over blind review without algorithm-annotations (Figure 5). None of the 16 FP markers for Subject 9 were noted by the consensus review. None of the 6 TP or 99 FP markers for Subject 6 were noted by the consensus review, though one of the true events was found by consensus in the EEG record without algorithm-determined markers. Only one of the 131 FP markers for these subjects was noted as an electrographic seizure by just one of the reviewers overall. It's possible that the large number of algorithm-annotation markers for these subjects led to a disbelief by the reviewers that any of these markers were valid. It is well-known since Aesop that credibility of a detection system is intricately intertwined with sensitivity and false alarms. Effectiveness of the system depends on this credibility. Yet, each false alarm reduces credibility that in turn affects response to future alarms, known as the false alarm effect (28), or the “cry wolf effect.” Paradoxically, the more sensitive a system, the greater the credibility of the system is affected by the false alarm effect due to the false alarm rate. Furthermore, as the credibility of the system is reduced by the false alarm effect, the credibility of the danger simultaneously increases. This is especially the case for sparse events, such as low seizure rates.

The intended benefit of automated algorithm detections as CDSS is to reduce the time it takes an epileptologist to review long-duration recordings while improving the sensitivity for finding relevant electrographic seizures. It is possible and likely that, with epileptologist experience and trust in the algorithm, CDSS could reduce the time it takes to review the REMI montage, as well as improve the sensitivity for detecting electrographic seizure events.

### Automated Seizure Detection Software

The results show that the automated algorithm can detect discrete focal-onset seizures in the REMI montage with a mean sensitivity of 90% and mean FDR of 0.087 FP/h across all subjects, on par with the Persyst P12 predicate of 81% sensitivity and FDR of 0.21 FP/h. There are a wide variety of commercially- and clinically-available software for EEG seizure detection. The most widely used software is Persyst and their P12 software is the most common benchmark for predicate comparison. Persyst has FDA-clearance for their most recent P13 and P14 seizure detection software, where the most notable difference is in the FDR. Recent studies have shown the P13 algorithm to have similar sensitivity to the P12 clearance, but higher FDR [0.5 FP/h in Scheuer et al. (20) and 0.9 FP/h in Koren et al. (18)]. The very recently released P14 seizure detection software has been shown to again have similar sensitivity to both P12 and P13, but with a much reduced FDR [0.04 FP/h, Scheuer et al. (20)]. Other FDA-cleared EEG seizure detection software show similar performance characteristics to the predicate P12 software, including the Nihon Kohden QP-160AK with 77% sensitivity and 0.45 FP/h (22) and Encevis [75% sensitivity and 0.29 FP/h in USFDA (23) and 78% sensitivity and 0.2 FP/h in Koren et al. (18)]. The sensitivity and FDR of the REMI automated algorithm outperforms the P12 predicate characteristics and remains on par with the best results of the other clinical EEG seizure detectors. The overlap between known seizure timings and algorithm-determined events was generally very high, with 92.1% mean overlap (Table 4).

The automated algorithm missed three known seizure events, all Type IB—Focal with Impaired Awareness (one for Subject #11 and two for Subject #20). For Subject #11, the one missed event occurred because the integrated ictal likelihood never crossed the seizure event threshold during the known seizure event timing. While there was a spike in the integrated ictal likelihood at this time, the threshold would have to be lowered by ~3% for an event to be detected at the known event time, though this would lead to a much higher FDR across all subjects. For Subject #20 it is more difficult to speculate why the two events were missed. A few possible reasons exist in that both events were short in duration (<30 s) and both events were noted in the clinician notes and wired-EEG to have strong motor content and some of this was picked up in the Epilog sensor EEG, especially in the sensor located at the right forehead. Thus, the duration of electrographic signal in the REMI montage may have been very short compared to movement-related artifact. None of the individual blinded reviewers noted seizures at the time of these three events.

**Figure 7:**
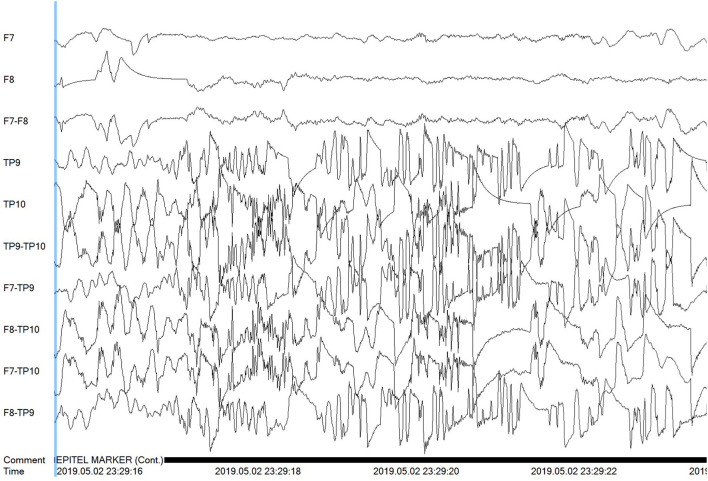
Poor signal quality is shown for the Tp9 and Tp10 Epilog sensors on Subject #9 during a false positive algorithm detection event.

Subjects 4, 6, and 9 can be considered outliers based on their much higher rate of FP when compared to the other 17 subjects. This is especially evident for the 99 FP found by the automated algorithm for Subject #6. Subjects 4 and 6 were known to have focal-onset seizures with impaired awareness, both with right hemisphere and mainly temporal localization. While not shown here, some of the algorithm-detected FP events for these subjects look very similar to known true events in evolution and localization. In the clinical notes, subject 6 was noted to have “several seizures many of which were subclinical and short in duration.” Six of these events occurred while the subject was wearing Epilog sensors and the subject had an additional five events outside the timeframe when the Epilog sensors were worn. It is possible that some or many of the FP events were short duration subclinical events that did not reach the initial expert reviewer's definition of a seizure and were not included in the seizure report. For Subject 9, whose wired-EEG “did not show any abnormalities,” there were a couple of FP events that look similar to known seizure events in other subjects, but most of the 16 FP events appear to be because one or more of the Epilog sensors were recording data with poor signal quality for unknown reasons (see channels TP9 and TP10 in Figure 7).

There are a number of ways that the REMI automated algorithm can be improved. A larger training data set will improve any bias or variance that the small data set used here contained. Larger training data sets can be created by enrolling more subjects in future studies as well as using data augmentation methods on current training data. Artifact rejection (e.g., muscular EMG contamination) and poor signal quality rejection can be added to the detection algorithm to improve the training data ground truths and seizure detection FDR.

### Ruling-In Electrographic Seizures

It is very common for someone to have no epileptic events during their EMU stay, simply because their epileptiform activity is rare and may not occur during a brief EMU stay. Of note are people who experience psychogenic non-epileptic seizures (PNES) that account for ~25% of all patients who enter the EMU (29). One potential application of REMI would be home monitoring for people experiencing seizures before an EMU visit and diagnosis. In this system, a person could wear Epilog sensors as part of REMI for long durations during their normal daily lives where the data could be remotely analyzed for electrographic seizures. This could help as an early mechanism to provide a better understanding of a patient's EEG before a costly, time-consuming, and possibly unnecessary visit to an EMU. If an EMU stay is warranted, REMI could be used to establish chronicity to an individual patient's electrographic events such that scheduling of the EMU stay could coincide with a higher probability of having a seizure (30, 31). When looking at their ability to rule-in subjects who experience electrographic seizures, the consensus review noted at least one TP seizure event for 9 out of the 10 subjects who had a known seizure event in their record (90% sensitivity). Similarly, the consensus review noted no events for 9 out of the 10 subjects who did not have any noted events in their record (90% specificity). The automated algorithm was tuned for high sensitivity and detected at least one true seizure event for all 10 known seizure subjects (100% sensitivity) and did not detect any false events for 7 of the 10 subjects that did not experience any seizures (70% specificity). For those who are experiencing very sparse epileptiform events, having an at-home, longer-duration EEG recording (as may be provided by REMI), would allow for more efficient and cost-effective electrographic diagnostics to rule them in for electrographic seizures.

## Limitations

This study was limited to EEG seizure data only from subjects who had focal-onset seizures. Focal-onset seizures (ILAE Type I) account for ~56% of all seizure types (32). Because focal-onset seizures may only show electrographic seizure activity in a very small region of the brain, their EEG correlates may not be captured by reduced-channel EEG systems, though some of these seizures evolve into bilateral tonic-clonic events where the electrographic activity becomes generalized across most regions of the brain (17% in this study). REMI is limited to four Epilog sensor placements (eight electrodes) on the scalp below the hairline, bilaterally on the forehead and behind each ear. The current clinical practice of high-channel-count wired electrodes is done to ensure complete spatial coverage of all brain regions and increase the likelihood of being able to differentiate very focalized epileptiform activity. The International Federation of Clinical Neurophysiology has recently recommended a minimum of 25 electrodes and up to 256 if necessary, in both adults and children (33), whereas the American Clinical Neurophysiology Society recommends a minimum of 16 channels (34). REMI does not have the spatial coverage of high-channel count systems and therefore may not be appropriate for seizure onset zone localization. However, EEG recorded from these four locations have been shown to detect 100% of electrographic seizures of both focal and generalized onset (35) due to volume conduction. The REMI montage may be useful for long-term seizure detection and chronicity. It will be important to show that both expert review of the REMI montage and use of detection algorithms can provide electrographic seizure detection in a broader range of seizure types, including those that have a generalized-onset.

Perhaps the greatest limitation of this study was that none of these reviewers had ever seen or reviewed Epilog EEG in the 10-channel REMI montage in the past, and longer-term experience and/or training may improve results. There were a few FP found and false negatives missed by all three reviewers and the automated algorithm. Epileptologists are trained to review video-EEG from EMU patients for the purpose of diagnosing seizure disorders. While these experts are well-trained at reviewing EEG, they can miss some seizures during standard waveform review (26, 36). In most studies on seizure detection, three or more expert reviewers are often relied upon to determine a consensus “ground truth” set of seizure events in the EEG record, and this was not done for the wired-EEG in this study. It may have been advantageous to review the wired-EEG for the FP found and false negatives missed here by the detection algorithm and blinded reviewers. However, EMUs do not typically keep complete wired-EEG records for more than a few months due to their large file size and storage limitations, and the complete wired-EEG records for these subjects were no longer available during this analysis.

There were only a limited number of subjects (20) and seizure events (24) used in this study. Better statistical power would be achieved through a much larger dataset. The long-term intent for the REMI system is for use in a person's everyday environment, yet the data studied here was collected only in the EMU, where subjects and the environment are very controlled. It will be necessary to show that the accuracy of review and software remains consistent in EEG recorded with REMI during a person's normal daily life.

The preferred method for this type of study would be to have a single, locked algorithm that was previously trained on a large independent data set, and then validated on all 20 of the patient's data included here. The EEG used in this study are the only data sets currently available where patients wore four Epilog sensors concurrently. Thus, the only way to train the automated algorithm was to use the largest data set available, which for each of the 20 patients was the independent data from all other 19 patients. A four-fold validation scheme was initially considered, instead of the 20-fold validation done here, where randomized data from five patients is held out of training, the training data would come from the remaining 15 patients, and then a single algorithm would be validated on five independent patient's data. The decision was made to not do this because it is possible and likely that a large number of seizure events would be held-out from one or more of the four-folds, likely making that fold's algorithm ineffective. Future work will include a single, locked algorithm trained on a large data set, and then validated on a separate set of multiple independent patients. It can be expected that those results would fall somewhere in the range of results described herein.

## Future Work

There are multiple ongoing and planned studies to continue the work described herein. Most importantly, a broader set of data encompassing all seizure types is currently being collected through collaboration with multiple clinical centers. This will allow a more rigorous analysis of which seizure types can be accurately reviewed from Epilog sensor EEG in the REMI montage. Collection and storage of the complete wired-EEG is a key part of these ongoing studies, so that any FP can be re-reviewed later from the wired record. The automated seizure-detection algorithm development is ongoing and future work involves expansion for all seizure types and any intricacies that their differences entail (e.g., absence seizures are very different in evolution and duration and how the algorithm handles these requires more complexity). Because Epilog sensor data can be captured over long durations, it will be critical to determine how long it takes for an epileptologist to blindly review the data, and if automated algorithm-detection annotations can reduce that time without affecting performance. Future studies will compare the automated algorithm performance on the REMI montage with FDA-cleared detection software that will be run on the simultaneously acquired wired-EEG. The Epilog sensors used in this study are intended as single-use, and the current study allowed for up to 7 days of Epilog sensor wear in the EMU. The ambulatory version of the Epilog sensor uses a rechargeable battery that is designed to be recharged once daily. A person would have multiple Epilog sensors, allowing them continuous EEG recording throughout their daily life. There are upcoming studies where Epilog sensors will be worn alongside AEEG systems in home environments to demonstrate the intended effectiveness of REMI for use in a person's normal daily life. Additionally, there are upcoming studies were Epilog sensors will be worn for months in the home environment to demonstrate the intended long-term effectiveness with a rechargeable version of the sensors.

## Conclusion

Epileptologists, without any REMI training or prior experience, reviewed EEG from just four Epilog sensors in the 10-channel REMI montage and accurately ruled-in subjects experiencing focal onset electrographic seizures with 90% sensitivity and 90% specificity. Consensus detection of individual spontaneous recurrent focal-onset seizures resulted in a mean sensitivity of 61% and mean FDR of 0.002 FP/h. Automated seizure detection algorithms used as Clinical Decision Support Software improved this sensitivity to 68% with little change to FDR (0.005 FP/h). The automated algorithm accurately ruled-in subjects experiencing electrographic seizures with 100% sensitivity and 70% specificity, and detected individual seizures with a very high mean sensitivity of 90% and low mean FDR of 0.087 FP/h, on par with current FDA-cleared software. Blinded epileptologist and automated algorithm review of the REMI montage showed strong potential to delineate patients who experience electrographic seizures. Such a system, when used in a person's everyday environment, could reduce the burden on EMUs to only those who truly need a differential diagnosis of a seizure disorder. Remote EEG systems and support software, as demonstrated here, will be critical to providing seizure diagnostic services to people in their normal daily lives, no matter where they live.

## Data Availability Statement

The raw data supporting the conclusions of this article will be made available by the authors, without undue reservation.

## Ethics Statement

The studies involving human participants were reviewed and approved by the University of Colorado, Colorado Multiple Institutional Review Board (COMIRB). The patients/participants provided their written informed consent to participate in this study. Written informed consent was obtained from the individual(s) for the publication of any potentially identifiable images or data included in this article.

## Author Contributions

MF created the automated algorithm, performed the statistical analysis, and is the main author of this manuscript. ML contributed to the experimental design, supported the algorithm development, and assisted in creation of this manuscript. MS coordinated the collection of all data from Epilog sensors alongside standard-of-care wired EEG recordings in the EMU. SR, BN, and AA were the reviewing epileptologists and assisted in creation of this manuscript. All authors contributed to the article and approved the submitted version.

## Funding

This work was supported in part by a grant from the National Institute of Neurological Disorders and Stroke NS100235.

## Conflict of Interest

MF and ML have financial interest in Epitel, Inc. The remaining authors declare that the research was conducted in the absence of any commercial or financial relationships that could be construed as a potential conflict of interest.

## Publisher's Note

All claims expressed in this article are solely those of the authors and do not necessarily represent those of their affiliated organizations, or those of the publisher, the editors and the reviewers. Any product that may be evaluated in this article, or claim that may be made by its manufacturer, is not guaranteed or endorsed by the publisher.
